# The Metabolization Profile of the *CYP2D6* Gene in Amerindian Populations: A Review

**DOI:** 10.3390/genes11030262

**Published:** 2020-02-28

**Authors:** Luciana P. C. Leitão, Tatiane P. Souza, Juliana C. G. Rodrigues, Marianne R. Fernandes, Sidney Santos, Ney P. C. Santos

**Affiliations:** 1 Oncology Research Center, Federal University of Pará, Belém, Pará 66073, Brazil; colaresluciana@gmail.com (L.P.C.L.); xtatixsouza@gmail.com (T.P.S.); julianacgrodrigues@gmail.com (J.C.G.R.); fernandesmr@yahoo.com.br (M.R.F.); sidneysantos@ufpa.br (S.S.); 2 Laboratory of Human and Medical Genetics, Institute of Biological Science, Federal University of Pará, Belém, Pará 66077-830, Brazil

**Keywords:** Amerindians, *CYP2D6*, phenotype, genetic polymorphisms, metabolization profile, Native Americans

## Abstract

Background: the *CYP2D6* gene is clinically important and is known to have a number of variants. This gene has four distinct metabolization profiles that are determined by the different allelic forms present in the individual. The relative frequency of these profiles varies considerably among human populations around the world. Populations from more isolated regions, such as Native Americans, are still relatively poorly studied, however. Even so, recent advances in genotyping techniques and increasing interest in the study of these populations has led to a progressive increase in publication rates. Given this, the review presented here compiled the principal papers published on the *CYP2D6* gene in Amerindian populations to determine the metabolic profile of this group. Methods: a systematic literature review was conducted in three scientific publication platforms (Google Scholar, Science Direct, and Pubmed). The search was run using the keywords “CYP2D6 Amerindians” and “CYP2D6 native Americans”. Results: a total of 13 original papers met the inclusion criteria established for this study. All the papers presented frequencies of the different *CYP2D6* alleles in Amerindian populations. Seven of the papers focused specifically on Amerindian populations from Mexico, while the others included populations from Argentina, Chile, Costa Rica, Mexico, Paraguay, Peru, and the United States. The results of the papers reviewed here showed that the extensive metabolization profile was the most prevalent in all Amerindian populations studied to date, followed by the intermediate, slow, and ultra-rapid, in that order. Conclusion: the metabolization profiles of the Amerindian populations reviewed in the present study do not diverge in any major way from those of other populations from around the world. Given the paucity of the data available on Amerindian populations, further research is required to better characterize the metabolization profile of these populations to ensure the development of adequate therapeutic strategies.

## 1. Introduction

### 1.1. Rationale

The cytochrome P450 2D6 (*CYP2D6*) is a member of the cytochrome P450 gene family, a group of enzymes that is responsible for phase I metabolism and the elimination of a variety of endogenous substrates and a diverse array of drugs [1]. The *CYP2D6* gene is the most frequently studied member of the P450 gene family in clinical research [2]. While this enzyme represents only a small proportion (1.3–4.3%) of all hepatic Cytochrome P450 enzymes (CYPs), it is known to metabolize more than 20% of all the drugs processed in the human liver, including at least 160 therapeutic targets, including antidepressants, antipsychotics, antiarrhythmics, opioid analgesics, anticancer agents, and other drug classes [3].

The *CYP2D6* gene is in the Chr22q13.1 region, close to two non-functional pseudogenes (*CYP2D7* and *CYP2D8*), and has a vast number of polymorphisms [4]. Up to now, over 125 allelic variants of the *CYP2D6* gene have been documented (PHARMVAR-https://www.pharmvar.org/gene/CYP2D6). These variants modify enzyme activity in various ways, that can be classified in four phenotypic groups: poor metabolizers (PM), intermediate metabolizers (IM), extensive metabolizers (EM), and ultrarapid metabolizers (UM) [5]. These differences in enzyme activity may result in both inter-individual and interethnic variation, with the relationship between the CYP2D6 genotypes and phenotypes being of considerable importance for the determination of therapeutic strategies in clinical practice [6].

The ethnic profile of a population may play an important role in the differentiation of the drug metabolism capacity among its individuals. Around the world, different populations carry alleles that characterize distinct *CYP2D6* phenotypes that vary among ethnic groups and, in turn, geographic regions. Llerena et al. (2014) reviewed the allelic variability of *CYP2D6* in major geographic regions and discovered that CYP2D6*4 (an allele with inactive enzyme activity, which is present in PM phenotypes) is most frequent in Europe [7]. Alleles associated with decreased enzyme activity are frequent in Asia and East Asia (CYP2D6*10), Africa and Afrodescedant populations (CYP2D6*17 and *29), with CYP2D6*41 and amplifications being found in Middle Eastern populations [7]. Zhou et al. (2017) analyzed the data available on the 1000 genomes platform, and offered considerable variability in several members of the CYP450 family, in particular *CYP2D6*, which varied greatly among the different populations for which data were available on the platform [8].

Although *CYP450* family genotypes and metabolic phenotypes have been studied extensively in different parts of the world [9,10], few data are available for some populations, such as those of the Native Americans (or Amerindians). The 2010 United States (US) Census [11] recorded a population of approximately 6.6 million Native Americans in this country. Worldwide, hispanics (including those from Latin America, the Caribbean, and the US) comprise a total population of more than 600 million individuals (http://data.worldbank.org/region/LAC), the equivalent of 8.4% of the world’s population. In addition, approximately 45 million Amerindians live in Latin America, representing 8.3% of the total population of this region (https://www.cepal.org/en/infografias/los-pueblos-indigenas-en-america-latina). 

Present-day Latin American populations are the end result of a process that began with migrations from northeastern Asia around 15,000–18,000 years ago, and was finalized over the past five centuries, following the arrival of Europeans and Africans, which led to extensive admixture [12,13]. Most New World populations reflect some degree of this process of admixture. The Mexican-American population is a multiple admixture of different ethnic groups, combining the genetic background of a number of Native American peoples, derived mainly from a single migration of Asians through Beringia, with white Europeans from Spain. There are approximately 70 groups of Amerindians in Mexico, with more than 85 languages and dialects, located mainly in the center and southeastern portions of the country, with an estimated total population of 10,113,411 [14]. Native Chileans, an important Amerindian population in South America, make up around 9% of the total population of Chile, that is 1,585,680 individuals who self-identified as Amerindian, according to Casen (2015) [15]. The Mapuche live in southern South America, in both Chile and Argentina, an area divided by the Andes, and account for approximately 84% of native Chileans [15,16].

In 2011, the population of Costa Rica was 4,301,712. This population is the result of admixture initiated during colonial times, which has blended Mesoamerican and European (primarily Spanish) genes with those from sub-Saharan African [17]. Approximately 2% of this population self-identified as Amerindian, distributed in different provinces all around the country, with distinct dialects and customs, including some individuals that do not belong to a tribe, but live in rural or urban areas [18]. In 2013, Paraguay had 19 indigenous tribes belonging to five linguistic families, with a total population of 112,848 individuals. In this country, indigenous peoples have been defined and grouped according to their relationship with the five language families, and are found throughout the country [19]. Like the other Latin American countries, Venezuela has undergone an intense process of admixture, and in the 2011 census, 724,592 individuals self-declared as native Americans. This population is distributed among eight indigenous communities, the Amazonas, Anzoátegui, Apure, Bolívar, Delta Amacuro, Monagas, Sucre, and Zulia [20].

In the 2007 census of Peru, a total of 1786 indigenous communities were identified and mapped in 11 of the country’s departments, with data being obtained on the populations and housing, with a total indigenous population of more than 4 million individuals [21]. In the United States, the Flathead Indian Reservation is home to three tribes, the Bitterroot Salish, the Upper Pend d’Oreille, and the Kootenai. The territories of these three tribes once covered all of western Montana and extended into parts of Idaho, British Columbia, and Wyoming [22]. Additional information on the ethnic groups and the countries in which they were studied is available in the Table S1. And Table 1 shows the published studies of the *CYP2D6* gene in Amerindian populations.

Many studies of American populations have demonstrated a degree of genetic heterogeneity in comparison with other ancestral populations, such as the Europeans and Africans, which also contributed to the formation of present-day New World populations [12,36,37]. The findings of these studies may contribute to the understanding of the interethnic variability of genetic polymorphisms in other populations, and their varying responses to drugs of clinical and therapeutic importance. In recent years, there has been considerable interest in research on the diversity of drug-metabolizing enzyme genotypes and phenotypes, in particular *CYP2D6*, in different populations, especially those of European origin [38,39]. Other population groups have also been the target of this research, albeit on a much smaller scale. In particular, relatively few studies have focused on Amerindian populations, to investigate the role of gene variants in the absorption, distribution, metabolism, and excretion of drugs.

### 1.2. Objective 

The present review aimed to compile the *CYP2D6* gene metabolization profiles of Amerindian populations through an extensive search of the available literature on molecular epidemiology, and to compare these data with those on other ethnic groups around the world.

## 2. Methods

### 2.1. Study Design 

**Figure 1:**
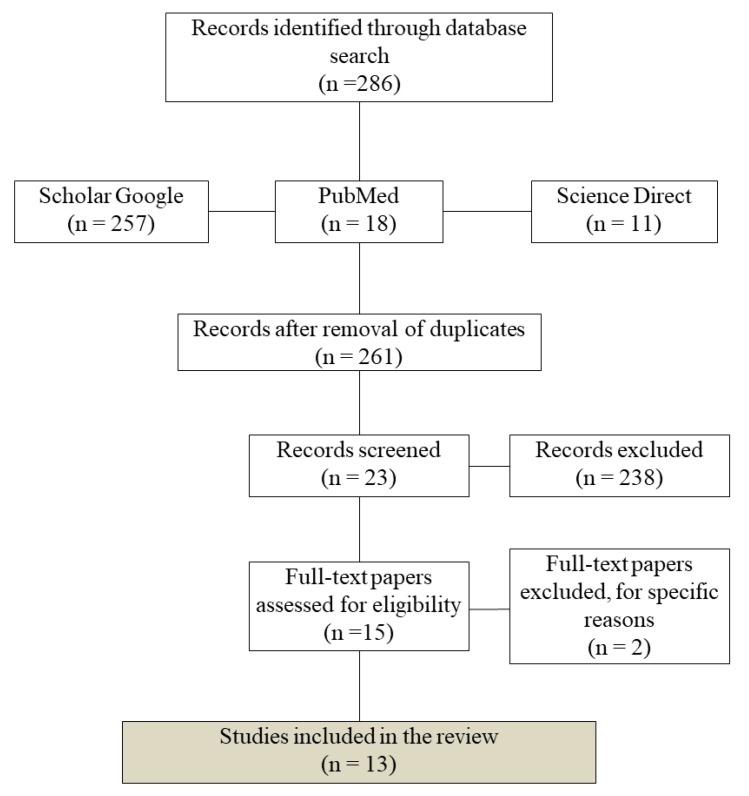
Flow diagram showing the number of records identified, included, and excluded from the present review.

A literature search was used to identify studies on three research platforms (Google Scholar, Science Direct, and Pubmed), considering the 20-year period between 1998 and 2018. The search was based on two key terms, “CYP2D6 Amerindians” and “CYP2D6 native Americans”, in all fields. The eligibility criteria were original papers in English, focusing on Amerindian populations, published between 1998 and 2018 (Figure 1).

### 2.2. Search Strategy

The following data were extracted from the selected papers: (i) the study population, (ii) the number of individuals analyzed, (iii) the polymorphisms of the CYP2D6 gene evaluated, and (iv) the allele and genotype frequencies. In all the papers selected here, the Amerindian populations were identified by self-declaration. The 1000 genomes platform was used as the reference database for the comparison of the allele frequencies of the most important gene mutations with those of other populations around the world.

## 3. Results 

A total of 13 studies were evaluated and included in the present review. All the papers addressed the frequencies of the different *CYP2D6* alleles in Amerindian populations. Seven of the papers focused specifically on Amerindian populations from Mexico, while the others included populations from Argentina, Chile, Costa Rica, Mexico, Paraguay, Peru, and the United States (Table 1). The Native American populations, their country of origin, and the *CYP2D6* alleles recorded in each study are identified in Table 1. The populations were grouped by country to facilitate the discussion of the observed patterns.

Some of the papers identified in the literature search did not present data on the genotyping of the metabolization profiles, but only their classification in the Active Score (AS) system. In the AS approach, each allele is assigned a value of 0 (non-functioning), 0.5 (decreased), or 1 (normal). For alleles with two or more gene copies, the value of the allele is multiplied by the number of copies (e.g., the duplication CYP2D6*1 × 2 is assigned an AS value of 2). The sum of the values assigned to the two alleles gives the AS score of the genotype. In this classification, an AS score of 0 was considered to represent a poor metabolizer (PM), while AS scores of 0.5–1 were classified as intermediate metabolizers (IM), scores of 1.5–2 as extensive metabolizers (EM), and scores of over 2 as ultrarapid metabolizers (UM) [40].

### 3.1. Synopsis of the Findings on the CYP2D6 Metabolization Profiles

#### 3.1.1. Poor Metabolizers

**Figure 2:**
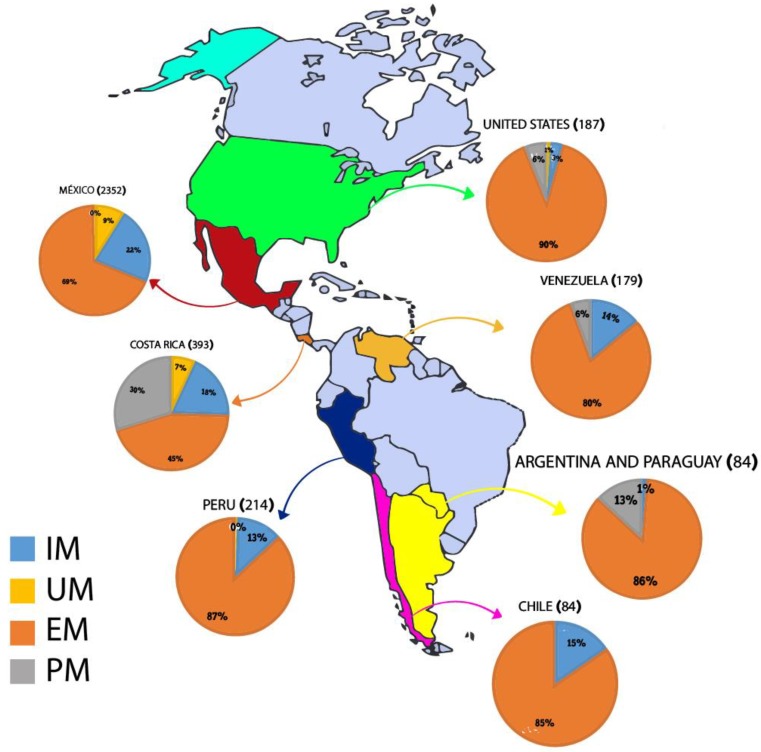
*CYP2D6* gene metabolization profile in Amerindian populations grouped by countries of America. IM: intermediate metabolizer; UM: ultrarapid metabolizer; EM: extensive metabolizer; PM: poor metabolizer.

Given the large number of polymorphisms found in the *CYP2D6* gene, the genotypes were characterized as the result of the interaction between haplotypes, with four principal metabolization phenotypes (Table 2). As the different variants have alternative functional consequences, individuals carrying these variants will have different levels of enzymatic activity. The metabolization profiles are classified according to the combination of alleles. The poor metabolizer (PM) profile is the result of the combination of two alleles that have a complete loss of function (no enzymatic activity), that is, null alleles due to mutations or the deletion of the gene [8,41]. The highest frequency of PM (30%) was recorded in Costa Rica [30,31], followed by Argentina/Paraguay, with 13% [34], and Venezuela [33] and the US [32], each with 6% (Figure 2).

The PM profile typically results in low levels of active metabolites of some medications, such as opioid analgesics, resulting in the reduced effectiveness of pain relief [42,43]. This indicates that individuals with the PM profile may require a modified therapeutic regimen or a follow-up for the diagnosis of the symptoms of insufficient pain relief [44]. 

#### 3.1.2. Intermediate Metabolizers

Individuals with the IM phenotype have a combination of two alleles with decreased enzyme function or the presence of one non-functional allele together with a second allele with decreased function [8,41]. The IM profile is the second most frequent (19%) in the Amerindian populations included in the present review. The highest frequency of the IM profile was recorded in Mexico (22%) [23,24,25,26,27,28,29,30], followed by Costa Rica (18%) [30,31], Chile (15%) [35], Venezuela (14%) [33], Peru (13%) [30], the US (3%) [32], and Argentina/Paraguay (1%) [34] (see Figure 2).

The therapeutic implications of this profile vary among different drugs. In the case of opioid analgesics, such as codeine, individuals with the IM profile form reduced quantities of morphine from the medication, requiring a follow-up during treatment, although no change in drugs is required [31]. Despite the effects of the alleles with reduced enzymatic function, few data are available on the clinical impacts on drug treatment, response to therapy, or side effects. 

#### 3.1.3. Extensive Metabolizers

The EM profile does entail any significant alteration in enzymatic activity. This profile may be determined, for example, by two alleles with normal enzyme function or the combination of one normal allele and one with decreased function [8,41].

The EM profile was the most common in all countries (Figure 2), and the Amerindian populations from different countries present relatively homogeneous frequencies of this profile. The highest frequency (90%) was recorded in the populations from the US [32], followed by Peru (87%) [30], Argentina/Paraguay (86%) [34], Chile (85%) [35], and Venezuela (80%) [33]. However, much lower frequencies were recorded in Mexico (69%) and Costa Rica (45%) [24,25,26,27,28,29,30,31].

It is important to note here that, while the EM phenotype typically has normal enzymatic activity, tests are still required for the more accurate prediction of the catalytic activity of this metabolization profile [45,46]. 

#### 3.1.4. Ultrarapid Metabolizers

Individuals with the UM profile carry at least one allele with increased enzyme function, in addition to an allele with normal function [41,47,48].

This profile was recorded in the Amerindian populations from three countries, Mexico, with a frequency of 9%, Costa Rica (7%), and the US, with 1% [24,25,26,27,28,29,30,31]. The highest frequency of this type of metabolizer has been recorded in an Ethiopian study (28.7%) [49]. Frequencies similar to those found in the Amerindian populations of Mexico have been recorded in Middle Eastern populations, i.e., 10.5% [7].

Despite the relatively low frequency of this profile in the study populations, its effects vary considerably, and may even be fatal. There have been a number of case reports of potentially fatal effects of standard doses of codeine in patients with the UM phenotype [50,51], which reinforces the need for the revision and alteration of the drugs used to treat these patients [52].

#### 3.1.5. Allele Frequencies 

**Figure 3:**
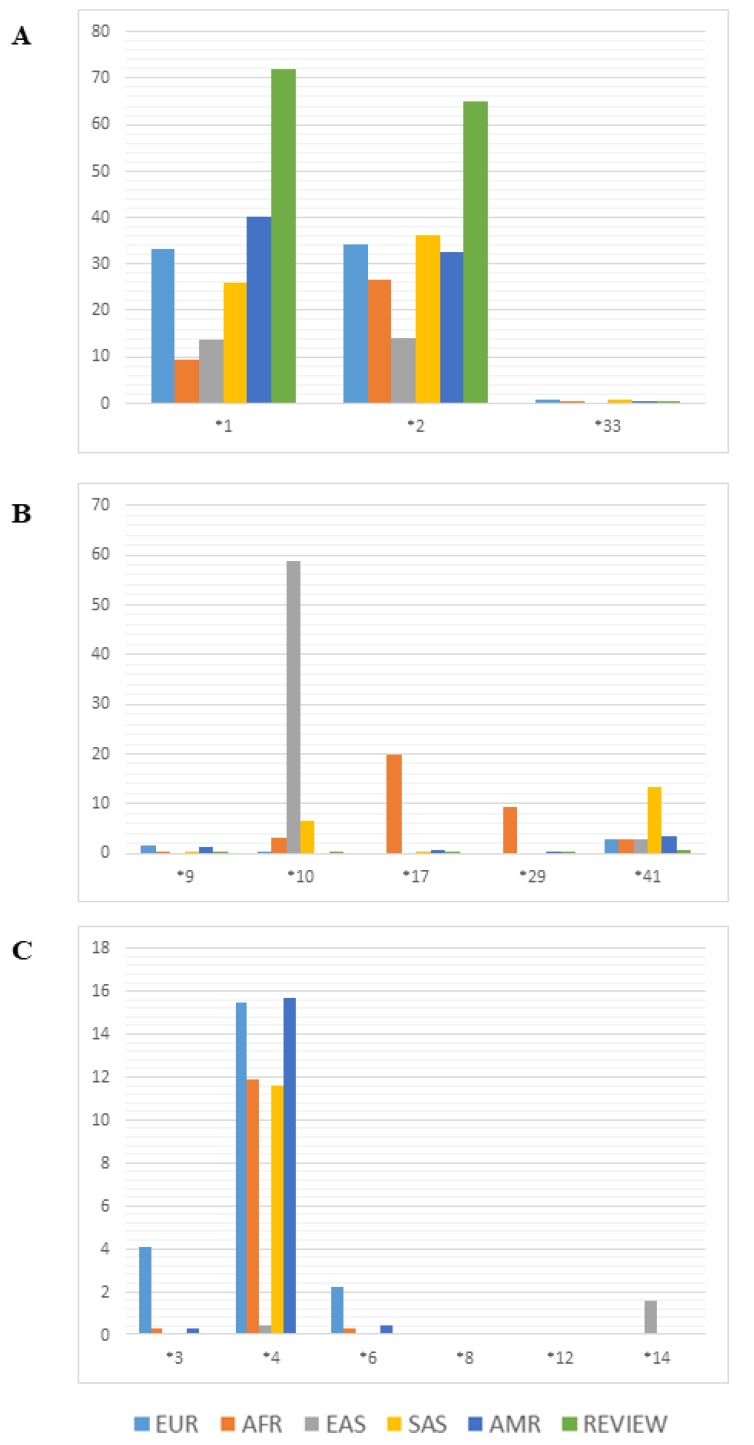
Allelic frequency of major variants of *CYP2D6* in world populations and in the Amerindian populations (data from review). (**A**) Variants with normal functional consequence; (**B**) variants with decreased functional consequence; (**C**) variants with inactivated functional consequence. EUR: European populations; AFR: African populations; EAS: East Asian populations; SAS: South Asian populations; AMR: American mixed populations; REVIEW: Amerindian populations from the review.

The frequencies of the principal alleles that alter the functionality of the CYP2D6 enzyme in the Amerindian populations analyzed in the studies reviewed here were compared with those of the same alleles in other human populations from different regions of the world (data obtained from the 1000genomes database). As the frequencies of the amplifications and deletions are not provided in the 1000genomes database, they are now reviewed here. These plots are divided according to the functional implications of the alleles (normal, reduced, or loss of function) for comparison with populations from Europe, Africa, East and South Asia, and the Americas (Figure 3).

The frequencies of the alleles with normal function are shown in Figure 3A. In general, the Amerindian populations have a high frequency of normal alleles, in particular *1 and *2, in comparison with the other populations. Admixed populations with a major Native American ancestry component have frequencies similar to those of the Amerindian populations presented here. Worldwide, 77–92% of individuals have at least one copy of a normal allele (*1 or *2) or two partially functional alleles [45]. 

The Amerindian populations presented frequencies close to 0% for all variants of the alleles with reduced function (Figure 3B). The CYP2D6*10 allele occurs at high frequencies in South Asian populations, while CYP2D6*41, which is caused by a splicing defect, has a frequency of less than 10%. The CYP2D6*17 allele, which results from two missense mutations, has a frequency of 20% in the African populations, while CYP2D6*29, which has four missense mutations, has a frequency of 10% in these populations.

The CYP2D6*4 variant, which is caused by a splicing defect, which inactivates the product of the *CYP2D6* gene, has frequencies ranging from 11.6% to 15.7% in most populations, except in East Asians and in the Amerindian populations reviewed here, in which the allele frequency was less than 1%. The frequencies of the other variants that cause the inactivation of the gene product were generally low, and did not vary greatly among populations (Figure 3C). However, CYP2D6*3 had a frequency of 4% in the European populations, while CYP2D6*14 had a frequency of 2%. The East Asian populations also presented frequencies of over 1% for the CYP2D6*6 variant. 

## 4. Discussion

The *CYP2D6* gene has many polymorphisms, resulting in a high level of inter-individual variability [8]. The molecular *CYP2D6* profile may alter the therapeutic efficacy of different drugs, which has led a number of international drug regulatory agencies to recommend the use of *CYP2D6* polymorphisms as biomarkers for the design of therapies based on antidepressants, antipsychotics, antiarrhythmics, opioid analgesics, anticancer agents, and other classes of drug [45,53]. A large number of the variants of the *CYP2D6* gene have important implications for the application of clinical therapy, and depending on their genomic profile, a patient may have a high risk of adverse reactions or even a failure of the therapy. The molecular profile of the *CYP2D6* gene varies considerably among populations [7]. 

The principal ancestral human populations—Europeans, Asians, Amerindians, and Africans—present considerable genetic diversity, which implies major fluctuations in the frequencies of important pharmacogenetic polymorphisms [54]. The traditional populations of the Americas have a long history of geographical isolation, and a differentiated genetic makeup in comparison with populations from other regions of the world [24]. The common origin of Native Americans and their autochthonous biological and cultural evolution combine to make the New World an excellent model for studies of the co-evolution between genes and cultures [54]. The papers selected for the present review permitted the compilation of the *CYP2D6* gene metabolization profiles of the Amerindian populations of a number of different countries in the Americas. The four metabolizing profiles (PM, IM, EM, and UM) had varying frequencies in the different countries (Figure 2).

The EM and IM metabolization profiles were the most frequent in all countries surveyed in the present review. Some drug regulatory agencies recommend the follow-up for patients using drugs metabolized by *CYP2D6* that have an IM profile. In the specific case of codeine, for example, there is evidence that some adverse effects do not vary significantly between poor and extensive metabolizers, which re-emphasizes the need for testing to determine the catalytic activity of individuals with these profiles to guarantee the best possible therapeutic strategy [42,45]. One important question here is that the extensive and intermediate metabolization profiles can be “converted” into a slow metabolization profile when exposed to other xenobiotics, such as alkaloid drugs or herbal medicines, which are known to be potent *CYP2D6* inhibitors, and are commonly used by Amerindian populations [48]. This is important given the high frequencies of the EM and IM profiles in these traditional populations and their extensive use of xenobiotics.

The ultrarapid metabolization (UM) profile has a relatively low frequency in the general Amerindian population (7%), although it is slightly higher in Mexican populations (9%) than in the other countries [24,25,26,27,28,29,30], probably due to the isolation of Mexican mestizos [24,30]. The UM profile was also substantial (7%) in Costa Rica [30,31]. These frequencies are similar to those described in other populations, such as that of Spain (6.1%) [50]. Individuals with this phenotype metabolize drugs faster than normal, which means that drugs taken at the standard dose may not have the intended therapeutic effects. Worse still, these individuals may also develop adverse reactions due to the formation of relatively large quantities of metabolites, between 10 and 30 times larger than normal [55,56].

The PM profile was relatively infrequent in the general Amerindian population (4%), a frequency similar to that recorded in Europe (6.52%) by Llerena et al. (2014) [7]. Low frequencies of slow metabolizers were recorded in a number of Native Mexican populations [23,24,26,27]. Sosa-Macías et al. (2010) found that Mexican Amerindian populations, in particular those of the Taphuanos community, had a more homogeneous slow metabolism profile distribution in comparison with Mexican mestizos [28]. This may be accounted for by the Tapehuano group and its low levels of miscegenation with Mexican mestizos. Individuals with the slow metabolizing phenotype have a potential risk of accumulating toxic metabolites when undergoing treatment with opioid analgesics, such as morphine and tramadol, and during long term therapy with antipsychotics and antihypertensive agents. In addition, they are prone to a lack of effective pharmaceutical action in the case of pro-drugs metabolized by the CYP2D6 enzyme, such as codeine and tamoxifen [29]. Given this, individuals of the Mexican Amerindian ethnic group have a lower risk of developing adverse drug reactions or therapeutic failure when treated with the aforementioned drugs.

The mean frequency of the alleles associated with the PM profile in Costa Rica, Argentina, Paraguay, the US, and Venezuela was 13.75%, with some variation being found among the Amerindian populations of these countries [30,31,32,33,34]. Naranjo et al. (2018) found that 10.2% of the indigenous Costa Rican population were slow metabolizers [30], a similar percentage to that recorded by Céspedes-Garro et al. (2014) for the same ethnic group [31]. In a survey of Venezuelan Amerindian populations, Grimam et al. (2012) found slow metabolizers in only one of the five populations analyzed [33]. Based on data from Argentinian and Paraguayan populations, Bailliet et al. (2007) concluded that some of the mutations that make up the PM genotype are founder variants brought to America by the early Asian settlers [34]. The different alleles of the slow metabolism profiles are described as important therapy predictors by Fohner et al. (2013), who evaluated native populations from the US, and suggested that CYP2D6 activity may be decreased (with high frequencies of the non-functional CYP2D6*4 and CYP2D6*41 variants) in 9.09% of the patients from the Salish and Kootenai tribes [32].

The results of the present review indicate that the frequencies of some of the alleles present in the Amerindian populations are similar to those of populations from other regions of the world (Figure 3). The majority of the *CYP2D6* alleles are shared by most of the world’s populations. Even so, a number of different evolutionary factors may have contributed to the establishment of geographical gradients in the distribution of some alleles, which occur at high frequencies in certain, specific regions of the world [57]. During a period of population expansion, in particular the initial wave, some rare alleles or haplotypes may become relatively common through founder effects [58].

A number of factors may be associated with the variation in allele frequencies of drug-metabolizing enzymes, such as CYP2D6, and the resulting metabolization profiles of Amerindian populations. Nebert (1997) identifies two possible selective pressures that may have determined this variation—differences in diet, which evolved over thousands of years, and the evolution of balanced polymorphisms, including alleles that confer resistance to bacterial or viral infections [59]. Considerable research efforts have been invested in the identification of predictive markers of therapeutic conduct for the development of personalized treatment protocols. In general, Amerindian populations have a different genetic profile than those of other populations around the world, which reinforces the need for specific studies of these ethnic groups for the identification of novel biomarkers relevant to the standardization of therapeutic strategies. The present review compiled the major publications available on the *CYP2D6* gene in Native American populations. As there is a clear under-representation of this ethnic group in pharmacogenomic studies, it will be essential to determine the *CYP2D6* profiles of a much larger number of Native American populations to support the development of systematic public health strategies.

It is important to note that substrate specificity and drug dosage are only two of a range of factors that contribute to the response of an individual to a given drug. Research groups, such as the Ibero-American Network of Pharmacogenetics and Pharmacogenomics (RIBEF) consortium, were established for the study of pharmacologically important genes in Latin American populations. The present review represents a pioneering compilation of the results of the available papers on the variation in the *CYP2D6* gene in Amerindian populations, and highlights the importance of this research for the development of guidelines for the management of therapeutic strategies in these populations. The major deficiency of the available papers is the lack of data from some countries, most notably, Brazil, Canada, and Colombia. These considerations reinforce the need for further, more extensive studies on the pharmacogenetics of Amerindian populations.

## Figures and Tables

**Table 1:** The published studies of the *CYP2D6* gene in Amerindian populations compiled in the present study.

Variants	Population, Tribe, or Affiliation	Country	Reference
*1 *3 *4 *6, *7, *8	Tarahumaras, Purépechas, Tojolabales, Tzotziles, and Tzeltales	Mexico	[23]
*1, *2, *4, *5, *6, *10, *3, *17, *29, *35, *41	Mexicaneros, Seris, Guarijíos, Tepehuanos, Mayos, Huicholes, Tarahumaras, and Coras	Mexico	[24]
*2, *3, *4, *6, *10, *17, *29, *35, *41	Tarahumara, Tepehuana, Mexicanera, Huichol, Cora, Seri, Mayo, and Guarijía	Mexico	[25]
*3, *4, *6, *10	Tepehuano	Mexico	[26]
*2, *3, *4, *5, *6, *10, *17, *35, *41	Lacandones	Mexico	[27]
*5, *2A, *35, *41	Tapehuanos	Mexico	[28]
*2, *3, *4, *6, *10, *17, *35, *41	Tzotzil and Tzeltal	Mexico	[29]
*2, *3, *4, *5, *6, *10, *17, *29, *35, *41	Bri bri, Cabecar, Chorotega, Guatuso, Guaymi and Huetar; Chol, Huichol, Lacandon, Mayo, Mexicanero, Seri, Tarahumara, Tepehuano, Tzeltal, Tzoltil, Yaqui and Zoque; Ashaninka, Aymara, and Shima	Costa Rica, Mexico and Peru	[30]
*1, *2, *3, *4, *5, *6, *10, *17, *29, *35, *41	Bribri, Guaymi, Cabecar, Guatuso, Chorotega, and Huetar	Costa Rica	[31]
*1, *2, *3, *4, *5, *9, *10, *28, *33, *35, *41	Salish and Kootenai	United States of America	[32]
*2, *3, *4, *5, *6, *10	Bari, Panare, Pemon, Warao, and Wayuu	Venezuela	[33]
*1, *2, *3, *4, *5, *6, *8, *10, *12, *14, *15	Jujuy province, Wichi, Chorote, Toba, Mapuche, Tehuelche, Ayoreo, and Lengua	Argentina and Paraguay	[34]
*1, *2, *3, *4, *5, *9, *10	Mapuches	Chile	[35]

**Table 2:** The different *CYP2D6* alleles recorded in the present review, and their respective variants and types of variation.

**Allele (Arranged by Functional Consequence)** **Normal Activity**	**Variants**	**Variation Type**
*1	None	-
*2	rs16947, rs1135840	Missense (R296C, S486T)
*33	rs28371717	Missense (A237S)
**Increased Activity**		
*1xN	Amplification of *1	-
*2xN	Amplification of *2	-
*53	rs1135822, rs1135823	Missense (F120I, A122S)
**Decreased Activity**		
*9	rs5030656	In-frame Deletion (K281del)
*10	rs1065852, rs1135840	Missense (P34S, S486T)
*17	rs16947, rs28371706	Missense (R296C, T107I)
*29	rs16947, rs1135840, rs61736512 and rs59421388	Missense (R296C, S486T, V136I and V338M)
*41	rs28371725	Splicing Defect
**Inactivated**		
*3	rs35742686	Frameshift
*4	rs3892097	Splicing Defect
*5	*CYP2D6* deletion	-
*6	rs5030655	Frameshift
*7	rs5030867	Missense (H324P)
*8	rs5030865	Stop gain (G169X)
*11	rs201377835	Splicing defect
*12	rs5030862	Missense (G42R)
*14	rs5030865	Missense (G169R)
*42	rs72549346	Frameshift
*62	rs730882171	Missense (R441C)

## Supplementary Materials

The owing are available online at https://www.mdpi.com/2073-4425/11/3/262/s1, Table S1: Population number and geographic location of the ethnic groups described in the review.
