# Supplementary material for: The Metabolization Profile of the CYP2D6 Gene in Amerindian Populations: A Review
Source: Genes (Basel). 2020 Feb 28;11(3):262. doi: 10.3390/genes11030262 (PMC7140882; doi:10.3390/genes11030262)
Supplement: Supplementary file 1 [file genes-11-00262-s001.pdf]

Supplementary Table 1. Population number and geographic location of the ethnic groups described in the review.

Country	Amerindian ethnic group	Population number	Geographic Location
Mexico*,**	Tarahumara	114.426	28.632295, -106.079051
	Purépecha	197.072	19.161708, -101.522507
	Tojolabales	54.348	16.552510, -92.972927
	Tzeltal	346.392	16.552510, -92.972927
	Tzotzil	429.024	16.552510, -92.972927
	Seri	666	29.988272, -110.918419
	Guarijio	2.567	25.958601, -107.048455
	Tepehuán	36.369	24.036793, -104.672104
	Cora	19.665	22.347239, -104.760214
	Mayo	102.709	30.330240, -110.522911
	Huichol	43.535	20.236990, -103.738758
	Lacandón	809	17.479066, -91.860172
	Yaqui	27.887	19.361883, -99.283330
	Zoque	78.622	16.994302, -93.280544
	Mexicaneros	540	24.028640, -104.654595
Chile***	Mapuche	1.329.450	37.853307, -71.659186
Argentina****	Jujuy province	-	24.182149, -65.308649
	Wichí	35.400	26.488773, -58.854651
	Chorote		21.988196, -62.337494
	Toba	46.000	36.852295, -65.293779
	Tehuelche		41.879474, -69.094110
Paraguay*****	Ayoreo	2.481	21.986866, -60.617536
	Lengua	-	25.278407, -57.661541
Venezuela	Bari	-	9.804807, -72.952929
	Panare	-	6.590384, -66.885177
	Pemon	-	4.884515, -61.504890
	Warao	-	8.063525, -63.848876
	Wayuu	-	11.441868, -71.976347
Costa Rica******	Bri bri	18.198	9.619880, -82.893601
	Cabecar	16.985	9.714659, -82.882844
	Chorotega	11.442	10.088065, -85.421051
	Guatuso	1.780	10.722945, -84.843341
	Guaymi	9.543	8.953380, -83.070533
	Huetar	3.461	9.869963, -84.236918
Peru*******	Ashaninka	88.703	8.978514, -73.178880
	Aymara	-	-
	Shima	-	-
United States	Salish and Kootenai	-	47.566629, -114.472763

*Boege, E. (2008). Regiones, territorio, lenguas y cultura de los pueblos indígenas. El patrimonio biocultural de los pueblos indígenas de méxico. Hacia la conservación in situ de la biodiversidad y agrodiversidad en los territorios indígenas, Eckart Boege (México: Instituto Nacional de Antropología e Historia-Comisión Nacional para el Desarrollo de los Pueblos Indígenas), 49-63.

** Solís, N P A. Mexicaneros - Pueblos Indígenas del México Contemporáneo (2007). México CDI, 46 p.

***Ministerio de Desarrollo Social. Casen 2015. Pueblos indígenas. Síntesis de resultados. http://observatorio.ministeriodesarrollosocial.gob.cl/casen-multidimensional/casen/docs/CASEN_2015_Resultados_pueblos_indigenas.pdf (accedido el 10/Abr/2018).
****ECPI. 2005. Encuesta Complementaria de Pueblos Indígenas: Ministerio de Economía y Produccioón/Secretaría de Política Económica—Instituto Nacional de Estadística y Censos. http://www.indec.mecon.gov.ar
*****Dirección General de Estadística, Encuestas y Censos (DGEEC) – III Censo Nacional de Población Y Viviendas para Pueblos Indígenas 2012. Available at: https://www.dgeec.gov.py/Publicaciones/Biblioteca/censo%20indigena%202012/Presentacion%20resultados%2019%2007%2013.pdf Data of access: 01/27/2020.
******Instituto Nacional de Estadística y Censos - Costa Rica (2012). X Censo Nacional de Población y VI de Vivienda 2011: Características Sociales y Demográficas Tomo II / Instituto Nacional de Estadística y Censos.--1 ed.San José, C.R. INEC. 340 p.
*******Instituto Nacional de Estadística e Informática (2009). Censos Nacionales 2007: XI de población y vi de vivienda - resumen ejecutivo resultados definitivos de las comunidades indígenas. Dirección nacional de censos y encuestas. 168p.
- The population number of those ethnic groups are not publicly available.
